# Monitoring and Management of Uric Acid Therapy in Gout and Chronic Kidney Disease: A Single-Center Retrospective Study

**DOI:** 10.7759/cureus.77813

**Published:** 2025-01-22

**Authors:** Ioannis Karageorgiou, Zeeshan Javed, Austen Grooms, Kamil Sardarli, Kostiantyn Romaniv, Julie George, Lisa Cohen

**Affiliations:** 1 Internal Medicine, William Beaumont University Hospital, Royal Oak, USA; 2 Internal Medicine, Oakland University William Beaumont School of Medicine, Rochester, USA; 3 Biostatistics, William Beaumont University Hospital, Royal Oak, USA; 4 Nephrology, William Beaumont University Hospital, Royal Oak, USA

**Keywords:** allopurinol, chronic kidney disease (ckd), gout disease, urate-lowering therapy, uric acid levels

## Abstract

Background

Gout commonly coexists with chronic kidney disease (CKD) due to reduced renal excretion of uric acid (UA). Guidelines recommend regular monitoring and dose adjustment of urate-lowering therapy (ULT), but the rate of adherence to these guidelines is not well established. Our study aimed to determine adherence to ULT guidelines in gout patients at our institution. In particular, we sought to assess the effect of CKD as well as other comorbidities on the prevalence of ULT guideline adherence.

Methods

We conducted a retrospective cohort study of 5,985 gout patients at our institution initiated on allopurinol between 2015 and 2020. Inclusion criteria were age over 18, a gout diagnosis, and a new allopurinol prescription. The primary outcome was UA monitoring within six months of therapy initiation. A secondary outcome was the prevalence of dose adjustments made by providers in response to a UA level above target.

Results

Only 48.3% (n = 2,889) of patients had UA levels monitored within six months. CKD stage did not significantly impact monitoring rates (p = 0.059). In patients with elevated UA levels (>6 mg/dL), 54.3% (n = 1,011) of patients had no dosage adjustments.

Conclusions

Significant gaps exist in adherence to ULT guidelines; nearly half of patients did not undergo recommended UA monitoring. Over half of patients with elevated uric levels did not have dosage adjustments. CKD stage did not affect the likelihood of UA monitoring or dose changes in persons with elevated UA levels.

## Introduction

Gout is a prevalent inflammatory arthritis caused by monosodium urate crystal deposition in the joint space. Its global burden is increasing, often accompanied by comorbidities such as chronic kidney disease (CKD) and cardiovascular disease [1]. Management focuses on urate-lowering therapy (ULT) to reduce uric acid (UA) levels. The 2020 American College of Rheumatology (ACR) and the 2016 European League Against Rheumatism (EULAR) guidelines advocate a treat-to-target UA level <6 mg/dL to prevent flares, resolve tophi, and reduce CKD progression risk [1-3]. Allopurinol, a xanthine oxidase inhibitor, is the first-line ULT agent. Traditional dosing calls for a reduced initial dosage of allopurinol in patients with CKD to minimize the risk of allopurinol hypersensitivity syndrome. There also exists a perception that allopurinol is nephrotoxic, although it is safe across the spectrum of kidney disease [4-9]. Several studies have shown that initial dosing of allopurinol across the spectrum of kidney function often leads to UA levels above the guideline target [4,10,11]. Nonetheless, adherence to ULT guidelines remains suboptimal [4,10,12]. Gout frequently coexists with comorbidities like hypertension, obesity, diabetes, and CKD, complicating treatment decisions [13]. Effective management requires an integrated approach addressing both gout and associated conditions [14]. Standardized care protocols and integrated management strategies are necessary, especially for CKD patients [15].

We sought to evaluate the prevalence of appropriate UA monitoring in patients with a gout diagnosis and a new prescription for allopurinol. Additionally, we looked at whether UA levels above target were met with an allopurinol dosage change. Finally, we assessed whether the presence of CKD influenced either monitoring of UA levels or appropriate dose adjustment in the setting of UA levels above the therapeutic target.

## Materials and methods

Study design, setting, and participants

We conducted a retrospective observational cohort study to evaluate adherence to ULT guidelines in gout patients with CKD. The study included patients diagnosed with gout and treated with allopurinol at William Beaumont University Hospital in Royal Oak, Michigan, USA, from January 2015 to December 2020. Inclusion criteria were patient age over 18 years, a diagnosis of gout, and receipt of a new prescription for allopurinol within the specified study period. The study excluded patients undergoing hemodialysis or peritoneal dialysis, those who received a solid organ transplant, patients diagnosed with cancer requiring systemic chemotherapy, those receiving azathioprine, and patients with AKI within three months of ULT prescription.

Data collection, variables, and outcomes

Data were extracted from electronic medical records and included patient demographics (age, ethnicity, and gender), presence of comorbidities (e.g., hypertension, coronary artery disease, and diabetes), and medications affecting UA excretion (allopurinol and febuxostat). UA level values were sought in the electronic medical record for each patient six months before and after the start of each allopurinol script and for every subsequent dosage change. The primary outcome was the measurement of serum UA levels within six months of starting allopurinol. The secondary outcome was whether the allopurinol dosage was changed in response to a UA level above target (>6 mg/dL).

Statistical analysis

Descriptive statistics include counts and percentages for categorical variables and means, SDs, medians, and ranges for continuous variables. Chi-square tests and t-tests were performed to examine the relationship between individual factors and UA monitoring outcomes. For analyses involving race and CKD stage, patients with missing data were excluded. Statistical analyses were performed using SAS 9.4 (SAS Institute, Cary, NC, USA). This study was approved by the institutional review board of William Beaumont University Hospital (IRB approval number 2022-120). Patient confidentiality was maintained following HIPAA regulations.

## Results

Characteristics of participants at baseline

The study included 5,985 patients prescribed allopurinol, with a mean age of 61.2 ± 14.2 years (median 61.0, range 22-105), as presented in Table 1. The majority were male (74%, n = 4,429) and White (65.9%, n = 3,944), followed by Black patients (24.3%, n = 1,455), those classified as “Other” races - which includes American Indian/Alaska Native, Asian, Native Hawaiian/Pacific Islander, and others (9.1%, n = 547) - , and “Unknown” race (0.7%, n = 39). Common comorbidities among the participants were hypertension (78.5%, n = 4,701), diabetes (35.4%, n = 2,116), coronary artery disease (19.5%, n = 1,165), and congestive heart failure (14.2%, n = 852). A subset of patients (18.2%, n = 1,088) had none of these comorbidities. Regarding CKD stages, 21.7% were at Stage I (n = 1,298), 43.3% at Stage II (n = 2,592), 16.4% at Stage IIIa (n = 979), 10.7% at Stage IIIb (n = 640), 4.4% at Stage IV (n = 265), and 1.2% at Stage V (n = 73); 2.3% (n = 138) had no glomerular filtration rate data available.

**Table 1:** Patient characteristics and rates of UA monitored Values are presented as numbers (percentages) unless otherwise specified. p-values reflect the results of chi-square tests comparing the proportion of patients with UA monitoring among the indicated subgroups. ^*^ For each comorbidity, the p-value represents a comparison between those with and without the condition. CKD, chronic kidney disease; GFR, glomerular filtration rate; UA, uric acid

Parameter	No. of patients (%)	% UA monitored	p-value
Patients on allopurinol	5,985	48.3	
Age			
Mean ± SD	61.2 ± 14.2		
Median (range)	61.0 (22-105)		
Sex			0.016
Female	1,556 (26)	50.9	
Male	4,429 (74)	47.3	
Race			0.001
Black	1,455 (24.3)	45.1	
White	3,944 (65.9)	48.5	
Other	547 (9.1)	54.8	
Unknown	39 (0.7)	53.8	
Comorbidities^*^			
Diabetes	2,116 (35.4)	47.8	0.573
Coronary artery disease	1,165 (19.5)	48.5	0.863
Congestive heart failure	852 (14.2)	50.5	0.166
Hypertension	4,701 (78.5)	48	0.371
None of the above	1,088 (18.2)	50.6	0.096
CKD stage			0.059
No GFR data	138 (2.3)	N/A	
Stage I	1,298 (21.7)	49.2	
Stage II	2,592 (43.3)	47.2	
Stage IIIa	979 (16.4)	51.8	
Stage IIIb	640 (10.7)	53	
Stage IV	265 (4.4)	47.9	
Stage V	73 (1.2)	47.9	

Primary and secondary outcomes

The primary outcome, measurement of serum UA levels within six months of starting allopurinol, was achieved in 48.3% of patients (Table 1). Monitoring rates differed significantly by sex (p = 0.016), with females having a higher rate (50.9%) compared to males (47.3%). Patients whose race was categorized as “Other” races had the highest monitoring rate at 54.8%, followed by “Unknown” race at 53.8%, White patients at 48.5%, and Black patients at 45.1%. (Of note, our patient population is comprised of a large Arab American population that would fall under the “Other” race category.). Comorbidities such as diabetes (p = 0.573), coronary artery disease (p = 0.863), congestive heart failure (p = 0.166), and hypertension (p = 0.371) did not significantly affect UA monitoring rates. Patients without any of these comorbidities had a monitoring rate of 50.6%, which was not statistically significant but approached the threshold (p = 0.096). CKD stage did not significantly influence monitoring rates (p = 0.059), although there was a trend toward higher rates in patients with Stage IIIa (51.8%) and Stage IIIb (53%).

For the secondary outcome (Table 2), among the 2,889 patients who had a post-initiation UA measurement (i.e., excluding the 3,096 patients for whom UA was “Not done”), 1,863 (64.5%) had a serum urate level >6 mg/dL. Of those with elevated UA, 54.3% (n = 1,011) did not undergo a dosage change, 35.9% (n = 668) had their dose increased, and 9.9% (n = 184) had their dose decreased (p < 0.001).

**Table 2:** Allopurinol dose change and UA levels Values are presented as numbers (percentages). p-values reflect the results of chi-square tests comparing dosage changes (no change, increased, or decreased) across the three UA categories. ^*^ Includes patients with only one script and dosage never changed UA, uric acid

Parameter	No change^*^ (%)	Increased (%)	Decreased (%)	p-value
UA (mg/dL)				<0.001
Not done	2,259 (73)	530 (17.1)	307 (9.9)	
Normal (≤6)	732 (71.3)	162 (15.8)	132 (12.9)	
Abnormal (>6)	1,011 (54.3)	668 (35.9)	184 (9.9)	

**Figure 1:**
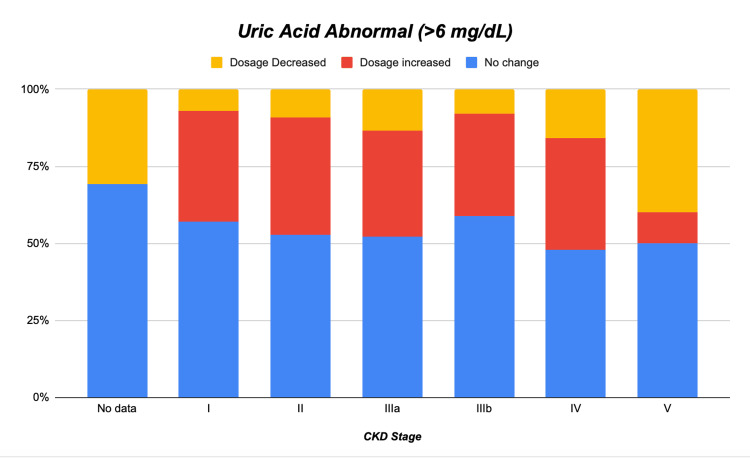
Distribution of allopurinol dose adjustments by CKD stage in patients with elevated UA (N = 1,863) This figure depicts the distribution of dosage adjustments among 1,863 patients with elevated UA levels (>6 mg/dL), stratified by CKD stage. Each stacked bar shows the proportions of patients whose allopurinol dose was unchanged (blue), increased (red), or decreased (yellow). CKD, chronic kidney disease; UA, uric acid

As shown in Figure 1, across all stages of CKD, the majority of patients did not have a dosage change, despite elevated UA levels. Dosage increases were the second most frequent outcome in every CKD stage, and dosage reductions were comparatively infrequent. Notably, this pattern appeared relatively consistent across CKD stages, indicating that neither dose escalation nor reduction was more prevalent in any particular CKD category.

## Discussion

Summary of findings

Our study revealed that slightly less than half (48.3%) of the 5,985 patients newly prescribed allopurinol for gout had serum UA levels checked within six months - a figure that underscores a notable gap in adherence to guideline - recommended monitoring. While monitoring rates were higher in female patients and those identifying as “Other” race, common comorbidities (diabetes, hypertension, coronary artery disease, and congestive heart failure) did not significantly alter the likelihood of follow-up testing. Interestingly, although CKD stage itself was not statistically associated with a higher probability of monitoring, there was a trend toward increased testing in Stages IIIa and IIIb, suggesting some physician awareness of the importance of tighter oversight in patients with moderate renal impairment. Among those who had post-initiation UA measurements, nearly two-thirds (64.5%) exhibited serum urate levels above 6 mg/dL, reflecting a considerable burden of undertreated hyperuricemia in this population. Despite elevated levels, a majority (54.3%) did not receive any dosage change. Although increases in allopurinol dose were more common (35.9%) than reductions (9.9%), both interventions remained underutilized relative to the proportion of patients whose UA exceeded guideline targets. Furthermore, the distribution of dosage adjustments was remarkably consistent across all CKD stages, suggesting that reservations about dose escalation may be pervasive and not solely driven by declining renal function. These findings collectively highlight suboptimal implementation of a treat-to-target strategy for gout care.

Comparison with existing literature

Our results corroborate previous work demonstrating that real-world adherence to UA monitoring guidelines remains insufficient [4,10]. While many studies in this domain have focused on veteran populations, our diverse cohort - encompassing both urban and suburban communities in the Detroit metropolitan area - extends these observations to a broader patient population. The low rates of UA monitoring and dosage modification seen here may stem from several factors. First, patients with gout typically carry multiple comorbidities, diluting clinical attention to hyperuricemia management and complicating decisions about dose escalation. Second, some clinicians may remain unfamiliar with updated recommendations emphasizing regular UA assessment and treat-to-target protocols. Finally, apprehension about adverse effects or misconceptions regarding allopurinol’s safety in CKD may deter more aggressive dose adjustments, even though recent evidence supports the safe use of allopurinol across various stages of renal dysfunction [4-9]. In line with the 2020 ACR guidelines and the 2016 EULAR recommendations, regular UA monitoring and titration of ULT to a target of <6 mg/dL are considered cornerstones of effective gout management [1-3]. Nonetheless, our data show that these principles are not consistently enacted in routine care. Emerging research has associated better urate control with decreased gout flares, reduced tophus burden, and less renal function decline [16]. Yip et al. (2020) questioned whether asymptomatic hyperuricemia is truly benign, given its associations with hypertension, cardiovascular disease, and CKD progression [17], but the topic remains contentious [18,19]. This challenges the traditional view and suggests that proactive management may be warranted to prevent long-term comorbidities. Our study’s low rates of UA monitoring and dosage adjustments indicate missed opportunities to address asymptomatic hyperuricemia as a potential contributor to CKD progression. Therefore, failing to adjust medication doses in response to persistently elevated UA levels represents a missed opportunity to optimize clinical outcomes. Our finding that neither comorbidities nor higher CKD stages significantly changed the likelihood of dosage escalation suggests that barriers to guideline adherence are multifactorial and not strictly tethered to clinical severity.

Although our primary analysis revealed that sex and race had significant roles in determining whether a patient’s serum UA was monitored within six months of initiating allopurinol, prior studies have also highlighted the influence of demographic factors - such as age or sex - on both the monitoring and adjustment of ULT. For instance, some investigations have reported that female patients may have higher engagement in healthcare and more frequent follow-up visits, which could partially explain increased monitoring rates compared to males, even though gout is generally more prevalent in men [14]. Our observation that “Other” races (including Asian, Native Hawaiian/Pacific Islander, American Indian/Alaska Native, and a notable Arab American population) and “Unknown” races had higher monitoring rates than Black or White patients may point to underlying practice or referral patterns at our institution or potentially greater health-seeking behavior in certain communities. Identifying the exact reasons for these disparities, however, requires more focused qualitative or community-based research. The relationship between age and gout management is another theme explored in earlier studies. Juraschek et al. (2015), for instance, found that older adults with gout were less likely to receive adequate ULT adjustments, in part due to concerns over polypharmacy, increased susceptibility to adverse drug reactions, and clinical inertia in patients with multiple comorbidities [12]. Although we did not specifically stratify dosage adjustments by detailed age categories, our overall findings that a significant proportion of patients - regardless of age - did not undergo dose modifications underscore the need for clinicians to balance safety considerations with the equally important goal of achieving optimal urate levels.

Potential interventions and future directions

Several strategies may help bridge this gap in practice. Automated prompts in electronic medical record systems can prompt providers to order a serum UA test upon prescribing or refilling allopurinol, ensuring that monitoring does not remain an afterthought. Targeted educational programs for physicians - focusing on guidelines, safe dose escalation, and the implications of uncontrolled hyperuricemia - may further improve rates of UA testing and intensification of therapy. Moreover, patient engagement initiatives, including reminders for laboratory assessments and enhanced education on the importance of maintaining a target UA level, could bolster overall adherence. Considering that gout flares often overshadow chronic management, an integrated approach ensuring follow-up testing once acute symptoms subside may further reinforce the importance of long-term ULT optimization.

Limitations

As a single-center, retrospective analysis, our study’s findings may be influenced by selection bias and may not be entirely generalizable to other settings, though the diversity of our patient population partly mitigates this concern. Since we relied on local electronic medical record data, UA tests performed at outside facilities could lead to an underestimation of true monitoring rates. Observational data also limit our ability to infer causality, leaving room for unaccounted confounding factors such as medication adherence, dietary habits, and socioeconomic determinants of health. Despite these limitations, our study offers a comprehensive view of real-world ULT management in a large cohort of gout patients with varying stages of CKD. The findings underscore ongoing gaps in guideline-based care - particularly around UA monitoring and dosage adjustments - that persist independently of renal function level. By shedding light on these deficiencies, this study provides a platform for targeted interventions designed to enhance gout management, reduce flares, and potentially safeguard renal health in this high-risk population.

## Conclusions

Our study highlights significant deficiencies in the management of gout among patients started on ULT therapy. Insufficient monitoring of UA levels persisted across various demographic factors and comorbidities, including the presence of CKD. Addressing these issues requires enhancing clinician awareness, integrating comorbidity management, and adopting individualized dosing strategies that consider factors beyond renal function alone. Improving adherence to ULT guidelines is essential to optimize patient outcomes and prevent disease progression.
